# Multidimensional Timbre Spaces of Cochlear Implant Vocoded and Non-vocoded Synthetic Female Singing Voices

**DOI:** 10.3389/fnins.2020.00307

**Published:** 2020-04-07

**Authors:** Molly L. Erickson, Katie Faulkner, Patti M. Johnstone, Mark S. Hedrick, Taylor Stone

**Affiliations:** Department of Audiology and Speech Pathology, University of Tennessee Health Science Center, Knoxville, TN, United States

**Keywords:** timbre, multidimensional scaling, cochlear implants, vocoding, singing voices

## Abstract

Many post-lingually deafened cochlear implant (CI) users report that they no longer enjoy listening to music, which could possibly contribute to a perceived reduction in quality of life. One aspect of music perception, vocal timbre perception, may be difficult for CI users because they may not be able to use the same timbral cues available to normal hearing listeners. Vocal tract resonance frequencies have been shown to provide perceptual cues to voice categories such as baritone, tenor, mezzo-soprano, and soprano, while changes in glottal source spectral slope are believed to be related to perception of vocal quality dimensions such as *fluty* vs. *brassy.* As a first step toward understanding vocal timbre perception in CI users, we employed an 8-channel noise-band vocoder to test how vocoding can alter the timbral perception of female synthetic sung vowels across pitches. Non-vocoded and vocoded stimuli were synthesized with vibrato using 3 excitation source spectral slopes and 3 vocal tract transfer functions (mezzo-soprano, intermediate, soprano) at the pitches C4, B4, and F5. Six multi-dimensional scaling experiments were conducted: C4 not vocoded, C4 vocoded, B4 not vocoded, B4 vocoded, F5 not vocoded, and F5 vocoded. At the pitch C4, for both non-vocoded and vocoded conditions, dimension 1 grouped stimuli according to voice category and was most strongly predicted by spectral centroid from 0 to 2 kHz. While dimension 2 grouped stimuli according to excitation source spectral slope, it was organized slightly differently and predicted by different acoustic parameters in the non-vocoded and vocoded conditions. For pitches B4 and F5 spectral centroid from 0 to 2 kHz most strongly predicted dimension 1. However, while dimension 1 separated all 3 voice categories in the vocoded condition, dimension 1 only separated the soprano stimuli from the intermediate and mezzo-soprano stimuli in the non-vocoded condition. While it is unclear how these results predict timbre perception in CI listeners, in general, these results suggest that perhaps some aspects of vocal timbre may remain.

## Introduction

Many post-lingually deafened adults who use cochlear implants (CIs) report that they no longer enjoy listening to music, and poor music perception is often reported as a significant negative factor in self-reported quality of life (Migirov et al., 2009). Cochlear implant signal processing favors the encoding of speech cues and allows users to perceive speech remarkably well using limited spectral and temporal acoustic information (Limb and Roy, 2014). While CI listeners may perceive speech well, some acoustic factors related to the perception of vocal timbre may not be adequately represented in the CI signal.

CI users have difficulty with many aspects of music perception. While rhythm cues are mostly preserved, CI users show deficits in the perception of pitch, melody, and timbre (Limb and Roy, 2014; Drennan et al., 2015; Jiam et al., 2017). Timbre is defined as that auditory attribute that distinguishes two sounds of equal pitch and loudness (ANSI, 1973). This definition must be modified a bit when discussing vocal timbre, which is that auditory attribute that distinguishes two vocal sounds of equal pitch and loudness that are also of approximately the same vowel. Vocal timbre is a perceptual attribute that is related to the acoustic characteristics of the output vocal signal and, therefore, is a function of the interaction of the glottal excitation source with the vocal tract transfer function (Cleveland, 1977; Sundberg, 1994, 2013; Roers et al., 2009).

Perceptually, differences in glottal excitation source spectral slope are believed to be related to the vocal quality dimension of *fluty* vs. *brassy* (Sundberg et al., 2004), while differences in overall resonance frequencies of the vocal tract have been shown to predict perception of Western classical voice categories such as mezzo-soprano and soprano (Cleveland, 1977; Dmitriev and Kiselev, 1979; Erickson, 2004). A clustering of the 3rd, 4th, and 5th resonances, known as the singer’s formant cluster (Sundberg, 1974), is associated with perception of *ring* in the voice (Ekholm et al., 1998) and may be related to behavioral modification of vocal tract configuration in either the hypopharyngeal or epilaryngeal area (Sundberg, 1974; Mainka et al., 2015; Story, 2016).

Physiologically, singing voice production often differs greatly from speaking voice production, resulting in differences in timbre between the two modes of voice use. In singing, physiological changes in glottal excitation source, vocal tract length (VTL), and non-vowel related shape of the vocal tract can occur within any given singer based on numerous factors. A detailed description of these factors is beyond the scope of this paper; however, as a starting point, the reader is directed to Johan Sundberg’s chapter in *The Psychology of Music* (Sundberg, 2013). Generally, these factors may be described as (a) variations across pitch and loudness (Echternach et al., 2016), (b) variations based on singing style (Sundberg et al., 1993, 1999; Thalén and Sundberg, 2001; Stone et al., 2003; Björkner, 2008; Borch and Sundberg, 2011; Bourne and Garnier, 2012; Guzman et al., 2015; Sundberg and Thalén, 2015; Yang et al., 2015; Bourne et al., 2016; Hallqvist et al., 2017), (c) variations based on vocal register (Titze, 1994; Sundberg and Kullberg, 1999; Sundberg and Högset, 2001; Roubeau et al., 2009), and (d) variations based on the need for a singer’s formant cluster (Sundberg, 1974, 1994, 2001, 2013; Dmitriev and Kiselev, 1979; Bloothooft and Plomp, 1986; Barnes et al., 2004; Johnson and Kempster, 2011; Mainka et al., 2015; Story, 2016). Thus, while speakers may keep a relatively constant glottal excitation source spectral slope and exhibit relatively small variations in VTL during speech, successful professional singers must learn to purposefully modify both the glottal excitation source and the vocal tract filter, resulting in vocal productions that are physiologically, acoustically, and perceptually much different from those of speech in many cases. Singers learn to modify both the excitation source of their instrument and the shape of their instrument in order to (a) produce a timbre that is consistent with the desired singing style, and, for many styles, (b) enable the production of pleasing timbre across pitch.

Research examining how well CI users perceive vocal timbre has not been focused on singing voice perception, but instead has focused on speaking voice perception with special attention to talker or gender discrimination or identification. CI users have been shown to have difficulty discriminating speakers (Cleary and Pisoni, 2002; Vongphoe and Feng, 2005; Sjoberg et al., 2017) and, when there is overlap in fundamental frequency, gender (Fu et al., 2005). One aspect of vocal timbre concerns the perception of cues in the acoustic signal that are related to VTL. Recent research has shown that CI users exhibit deficits in their ability to extract VTL cues, which could be a factor contributing to poor speaker and gender identification (Kovačić and Balaban, 2009; Massida et al., 2013; Fuller et al., 2014; Gaudrain and Başkent, 2015; Gaudrain and Baskent, 2018; Zaltz et al., 2018) and could contribute to difficulties in singing voice timbre perception as well.

The primary source of information concerning VTL in singers comes from x-ray data of Western classical singers collected in Dresden during the 1950s (Roers et al., 2009) and by Dmitriev and Kiselev in the 1970s (Dmitriev and Kiselev, 1979). The Dresden x-ray data were collected with the larynx at rest; while the Dmitriev and Kiselev x-ray data were collected during singing. The Dresden data have been analyzed by researchers in Dresden and Stockholm (Roers et al., 2009) using the methods employed by Dmitriev and Kiselev. These researchers found that resting VTLs obtained from sopranos demonstrated a great deal of variability, ranging from just under 130 mm to just over 160 mm. On the other hand, the resting VTLs obtained from mezzo-sopranos demonstrated less variability, ranging from 145 mm to just over 160 mm. There was no statistically significant difference in resting VTL between the two groups. Resting VTL also did not correlate with body height. The data obtained by Dmitriev and Kiselev show a high degree of overlap in the singing VTL of mezzo-sopranos and central sopranos (167–183 mm vs. 168–185 mm, respectively), with only the high sopranos exhibiting much shorter VTLs (153–163 mm). If the central and high soprano data are merged, the variability in the singing VTLs obtained by Dmitriev and Kiselev becomes very similar to the variability in resting VTL observed in the Dresden data. Dmitriev and Kiselev also measured the frequency of “the high singing formants” that occur above 2 kHz and, similarly to the VTL data, observed overlap between mezzo-sopranos and sopranos; with only the high sopranos having distinctly higher upper formant frequencies.

When designing a timbre perception study, researchers can choose to implement an identification task and/or a discrimination task, depending on the goals of the study. Studies utilizing identification tasks in order to examine instrument timbre perception in CI users have found that, generally, when presented with a musical note or song performed on an instrument, CI users demonstrate reduced ability to correctly identify the instrument from either closed or open sets (Schulz and Kerber, 1994; Gfeller et al., 1998, 2002; McDermott, 2004; Looi et al., 2008; Kang et al., 2009). However, identification of a specific instrument requires semantic knowledge of the instrument and an understanding of how the semantic label relates to the acoustics of the instrument. Identification studies do not provide information concerning how well CI users may be able to utilize timbral cues to discriminate between instruments. Multidimensional scaling (MDS) studies, on the other hand, often employ discrimination tasks and allow for the mapping of perceptual spaces without requiring participants to have direct knowledge of semantic labels.

MDS has been used to map the perceptual timbre spaces of instruments (Grey, 1977; Iverson and Krumhansl, 1993; Krimphoff et al., 1994; McAdams et al., 1995; Marozeau et al., 2003; Handel and Erickson, 2004; Caclin et al., 2005) and singing voices (Bloothooft and Plomp, 1988; Erickson, 2003, 2008, 2016, 2020) in normal hearing (NH) populations. In general, MDS studies using real and synthetically constructed instrument tones have revealed that temporal envelope/attack-time (Grey, 1977; Krimphoff et al., 1994; McAdams et al., 1995) and spectral centroid (Grey and Gordon, 1978; Iverson and Krumhansl, 1993; Krimphoff et al., 1994; McAdams et al., 1995; Handel and Erickson, 2004) are the dominant cues for the perception of the dissimilarity of instruments by NH listeners. Additional dimensional correlates found in instrumental MDS studies include spectral fluctuation (Krumhansl, 1989) and frequency vibrato extent (Handel and Erickson, 2004). In singing voices, 1/3 octave spectra (Bloothooft and Plomp, 1988), spectral centroid from 0 to 5 kHz (Erickson, 2008, 2020), spectral centroid from 2 to 5 kHz (Erickson, 2003), and, at higher fundamental frequencies, spectral centroid from 0 to 2 kHz (Erickson, 2020) appear to provide cues useful in judging timbre dissimilarity in voices as does frequency vibrato rate (Erickson, 2003, 2008).

MDS has been used to assess the perception of instrument timbre in NH listeners using vocoded stimuli (Macherey and Delpeirre, 2013) and to assess the perception of instrument timbre in CI users (Kong et al., 2011; Macherey and Delpeirre, 2013). Kong et al. (2011) found that the MDS instrument space produced by pre-lingually and peri-lingually deafened CI users appeared to be most influenced by attack-time cues with spectral centroid cues being less reliable and potentially less salient. However, in a study that examined instrumental MDS dimensions generated by 4 groups (Migirov et al., 2009), NH listeners (Limb and Roy, 2014), NH listeners rating 4-channel vocoded stimuli, Jiam et al. (2017) NH listeners rating 8-channel vocoded stimuli, and (Drennan et al., 2015) post-lingually deafened CI listeners, Macherey and Delpierre (Macherey and Delpeirre, 2013) found similar MDS solutions for all four groups. Dimension 1 organized stimuli according to attack-time. Dimension 2 was correlated with spectral centroid. It should be noted, however, that the CI MDS solution accounted for a smaller amount of variance than did any of the NH solutions and that, contrary to expectations, CI listeners weighted the spectral centroid dimension more strongly and the attack-time dimension less strongly than normal hearing listeners. The results of these two studies suggest that CI listeners may be able to use cues such as attack-time and spectral centroid to discriminate some elements of instrumental timbre. How well these results would generalize to singing voices, which do not differ much in attack-time and which have spectral characteristics that may not differ as much as those found between major classes of instruments, is unknown.

For the current study, an 8-channel noise-band vocoder was used to simulate how CI sound processing alters the perceived timbre of synthetic female singing voices with vibrato for both lower and higher pitched stimuli. NH listeners were presented with both non-vocoded and vocoded synthetic stimuli to examine how their perceptual timbre space was affected by this simulation. It was hypothesized that at lower pitches, 8-channel vocoding would result in the loss of important spectral characteristics, resulting in alterations of the multidimensional perceptual space. However, it was also hypothesized that at higher pitches, the wide spacing of harmonics would cause an under-sampling of the vocal tract transfer function. This under-sampling could cause a lack of spectral peaks in both the non-vocoded and vocoded conditions, theoretically resulting in similar MDS representations in those two conditions.

## Materials and Methods

### Listeners

All listeners provided written informed consent using a procedure approved by the Institutional Review Board of the University of Tennessee, Knoxville. Listeners were recruited from students enrolled in introductory psychology courses at the University of Tennessee, Knoxville and from faculty and students in the University of Tennessee Department of Audiology and Speech Pathology. Listeners were recruited who met the following criteria: (a) bilateral hearing within normal limits (≥20 dB from 500 to 4000 Hz) (ASHA, 1990) and (b) 18 years of age or older. Listeners recruited from Psychology courses were awarded class credit for participating in the study. Psychology students can receive such credit by participating in a variety of studies as well as by writing papers on the topic of research design in lieu of participating in research studies. Thirty listeners were recruited for the experiment; however, one participant did not pass the hearing screening and was removed from the study, resulting in a final N of 29. There were 21 female and 8 male participants with a mean age of 20.17 years and an age range of 18–30 years.

### Stimuli

#### Non-vocoded Synthetic Vocal Stimuli

Non-vocoded synthetic vocal stimuli were generated using a digital source-filter synthesizer. The synthesis model was built using Aladdin Interactive DSP workbench (Hi-Tech Development, Stockholm, Sweden). Aladdin synthesizes at 16 kHz, so the resulting upper spectral limit was at the Nyquist frequency of 8 kHz.

**FIGURE 1:**
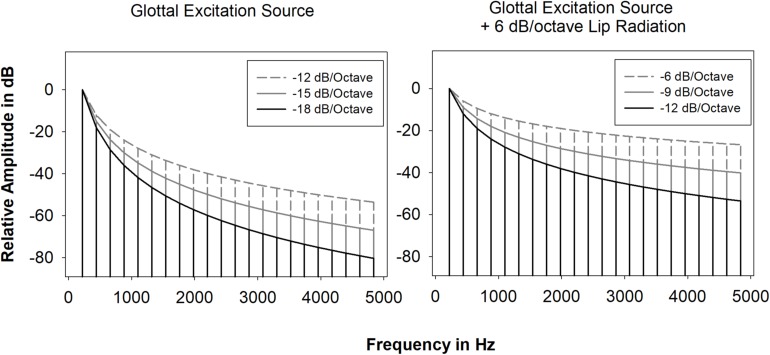
Glottal excitation source spectral slopes without and with +6 dB/octave.

For the pitches C4 (261.6 Hz), B4 (493.9 Hz), and F5 (698.5 Hz), signals to be used as input to the source-filter synthesizer (henceforth referred to as the excitation source) consisted of a number of harmonics equal to 8000 Hz divided by the fundamental frequency. These harmonics decreased in amplitude by 6 dB/octave, 9 dB/octave, and 12 dB/octave (Figure 1). The spectral slopes of these signals (excitation source spectral slopes) were calculated by adding lip radiation (+6 dB/octave) to glottal source spectral slopes that might be produced by female singers based on type or style of singing (12 dB/octave, 15 dB/octave, and 18 dB/octave). All stimuli were constructed with vibrato for the following reasons: (Migirov et al., 2009) due to the length of the study it would not have been possible to include vibrato and non-vibrato stimuli in the MDS analyses; (Limb and Roy, 2014) previous research utilizing non-vocoded and vocoded stimuli revealed nearly identical vibrato and non-vibrato MDS solutions (Jiam et al., 2017; Erickson and Faulkner, 2018); synthetic vibrato stimuli are much more naturalistic and less fatiguing than synthetic non-vibrato stimuli. Excitation source signals were synthesized using a frequency vibrato rate of 5.6 Hz and a frequency vibrato extent of ±50 cents (0.5 semitone). The vibrato rate and extent are values typical of Western classical singing (Hakes et al., 1987).

**FIGURE 2:**
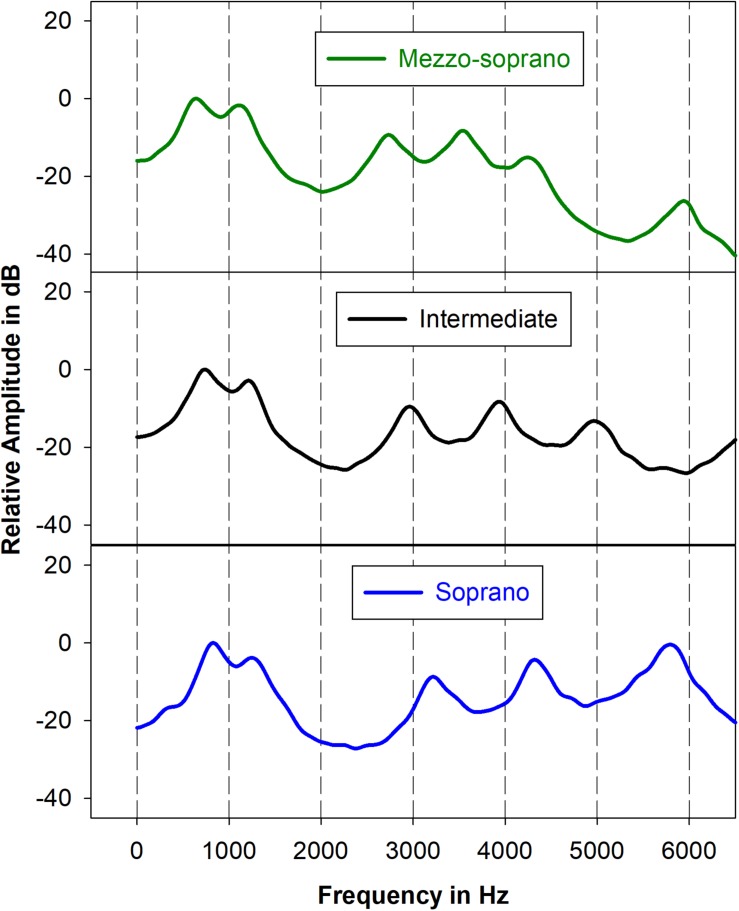
Mezzo-soprano, intermediate, and soprano transfer functions below 6500 Hz. Transfer functions were obtained by stimulating the source-filter synthesizer with white noise and smoothing the output.

Excitation source signals were filtered using 3 vocal tract transfer functions, M (mezzo-soprano), S (soprano), and I (intermediate) for the vowel/ɑ/ (Figure 2). Each transfer function was constructed using a cascade synthesizer and 8 resonance frequencies. Although 5–6 resonance frequencies would typically fall below the Nyquist frequency of 8 kHz, 8 resonances were used during the synthesis process because vocal tract transfer functions are the sum of overlapping vocal tract resonance filters and, therefore, the transfer function below 8 kHz can be affected by higher resonances. Resonance bandwidths were set to those used in a previous study (Erickson, 2004). Resonance frequencies for the transfer functions M and S were derived from an operatic mezzo-soprano and an operatic light coloratura soprano, respectively, using the following procedure: (Migirov et al., 2009) an 18-pole linear predictive coding (LPC) analysis at the pitch A3 was used to compute preliminary resonance frequencies for the first 8 vocal tract resonances then (Limb and Roy, 2014) resonance frequencies were modified, when necessary, through use of an analysis by synthesis procedure such that the resulting synthetic output spectral peaks corresponded with those of the original target stimulus at pitch A3. Comparisons of the original and synthesized spectra revealed that changes to synthesis bandwidths were not necessary. An intermediate (I) vocal tract transfer function was constructed by calculating intermediate resonance frequencies as follows:

(1) $$S\,T_{I}=.5\,\left(12\,l\,o\,g_{2}\,\frac{R_{S}}{R_{M}}\right)$$

(2) $$R_{I}=R_{M}\,\left(2^{S\,T_{I}/12}\right)$$

where *R*_*S*_ = the soprano resonance frequency, *R*_*M*_ = the mezzo-soprano resonance frequency, *ST_I_* = the number of semitones midway between *R*_*S*_ and *R*_*M*_ as measured in reference to *R*_*M*_, and *R*_*I*_ = the resulting intermediate resonance frequency. Resonance frequencies for vocal tract transfer functions M, I, and S are displayed in Table 1.

**TABLE 1:** Resonance frequencies for mezzo-soprano (M), intermediate (I), and soprano (S) stimuli.

Resonance	Frequency in Hz		
	
	M	I	S
1	625	712	811
2	1158	1227	1300
3	2725	2960	3217
4	3550	3915	4317
5	4300	4991	5793
6	5927	6789	7774
7	7732	8982	10,432
8	10,087	11,882	13,999

The synthesis procedure resulted in 9 stimuli for each of the following three conditions: C4 not vocoded, B4 not vocoded, and F5 not vocoded. Using Adobe Audition (Salt Lake City, Utah), each stimulus was edited to 1 s in duration and smoothed using spline curves applied to the onsets and offsets, and then normalized in average RMS amplitude. As with real voices, the spectral characteristics of the resulting non-vocoded stimuli were a result of the interaction of the systematically varied excitation source signal and the systematically varied vocal tract transfer function.

#### Vocoded Stimuli

To create the vocoded stimuli, the 9 stimuli from each of the 3 non-vocoded conditions were processed through an 8-channel noise-band vocoder using the AngelSim^TM^ Cochlear Implant and Hearing Loss Simulator (TigerSpeech Technology, Los Angeles, CA, United States). Input stimuli were filtered into 8 frequency analysis bands using fourth-order band-pass Butterworth filters, the cutoff frequencies of which were determined by a Greenwood function. The temporal envelope in each band was extracted using half-wave rectification and a low-pass fourth-order Butterworth filter with a cutoff frequency of 160 Hz. As with the analysis filters, there were 8 fourth-order band-pass Butterworth carrier filters, the cutoff frequencies of which were identical to the analysis filters. The filtered carrier noise in each band was modulated by the extracted amplitude envelope in the same band. It should be noted that this results in a broadening of each frequency band. The final modulated noise bands were summed to create the vocoded stimuli. Analysis and carrier filter parameter settings are listed in Table 2. The vocoding procedure resulted in 9 stimuli for each of the following three conditions: C4 vocoded, B4 vocoded, and F5 vocoded. Vocoded stimuli were normalized in average RMS to the non-vocoded stimuli. Due to the length of the study, it was not possible to include multiple vocoder configurations. The results of this study should be interpreted with that limitation in mind. Also, it cannot be said that a noise-band vocoder would accurately reflect the signal received and processed by CI users.

**TABLE 2:** Vocoder analysis and carrier filter parameters of lower cutoff frequency (F_*L*_), higher cutoff frequency (F_*U*_), and bandwidth (ΔF) in Hertz and semitones.

Band	F_*L*_ in Hz	F_*U*_ in Hz	ΔF in Hz	ΔF in semitones
1	200.0	359.1	159.1	10.13
2	359.1	591.3	232.2	8.63
3	591.3	930.5	339.2	7.85
4	930.5	1425.8	495.3	7.39
5	1425.8	2149.1	723.3	7.10
6	2149.1	3205.3	1056.2	6.92
7	3205.3	4747.7	1542.4	6.80
8	4747.7	7000	2252.3	6.72

### Experimental Design

Multi-dimensional scaling techniques were employed to determine the perceptual dimensionality of the non-vocoded and vocoded synthetic vocal stimuli. For each of the six conditions, the 9 stimuli were combined into all possible pairs, resulting in a total of 36 pairs for each condition for a total of 216 experimental pairs. Additionally, a practice experiment was created from 20 pairs that spanned a variety of combinations of the experimental stimuli, resulting in 236 stimulus pairs total. A within-subjects designed was used where each participant completed all conditions.

### Procedure

The listening experiment took place in a single-walled sound booth (Acoustic Systems RE-144-S, Austin, TX, United States). Stimuli were presented binaurally using Sennheiser HD 545 (Old Lyme, CT, United States) headphones. Prior to the practice session and experiment, listeners were told that they would hear two sounds and that it was their task to indicate how similar or different the two sounds were by using a scroll bar. They were told: (Migirov et al., 2009) if the two sounds were very different, they should drag the scroll bar toward the far right end (Limb and Roy, 2014); if the sounds were the same, they should drag the scroll bar all the way to the far left; and (Jiam et al., 2017) if the difference was somewhere between those two extremes they should drag the scroll bar to a corresponding location somewhere between the two ends. Listeners were warned that each stimulus pair would play only once with no opportunity to repeat the pair, so they should be prepared to listen closely for upcoming pairs.

Stimulus pairs in both the practice session and the subsequent experimental session were presented using MEDS (Music Experiment Development System) (UCLA, Los Angeles), an object-oriented development system designed by Roger A. Kendall (Windsor, 2004) that has been widely used in the construction and analysis of perceptual and psychoacoustic experiments. Due to the length of the experiment, stimulus pairs were presented once only. Using a 100-point scroll bar with endpoints labeled “Same” and “Very Different,” the listener’s task was to indicate the dissimilarity of the paired stimuli. Prior to the MDS experiment, each participant completed the practice session which was composed of 20 randomly ordered stimulus pairs systematically selected to include very similar and very different stimuli of non-vocoded and vocoded pairs across all three pitches. Participant performance on the practice task was monitored and if the researcher felt that the participant did not understand the instructions, the participant was reminded of the experimental task as well as of how to use the scroll bar. After the practice session, listeners completed the MDS experiment. Listeners were presented with counter-balanced blocks, one for each of the six conditions (C4 not vocoded, C4 vocoded, B4 not vocoded, B4 vocoded, F5 not vocoded, and F5 vocoded). This within-subjects designed allowed each participant to act as their own control. Within each block, the 36 pairs were presented in random order.

### Acoustic Measures

The current experiment employed several acoustic measures in order to identify those spectral cues that may correlate to specific MDS dimensions. The synthetic stimuli employed in this study have a fixed attack-time, so stimulus onset was not included as an acoustic variable. Because any spectral fluctuation would be a result of frequency modulation of the excitation source which was constant across all stimuli, this also was not included as an acoustic variable. In total, 4 acoustic measures were computed: spectral centroid from 0 to 8 kHz, spectral centroid from 0 to 2 kHz, spectral centroid from 2 to 8 kHz, and energy ratio. The method of calculation for each of these acoustic measures are described in the following sections. Each measure was calculated from the middle of the 1 s sample. All measures were made using the Fast Fourier Transform (FFT) algorithm provided by Praat (Paul Boersma and David Weenink, Institute of phonetic Sciences, University of Amsterdam, The Netherlands) using an analysis window of 0.75 s.

#### Spectral Centroid Measures

Spectral centroid, a measure of the weighted mean frequencies within a specified frequency range, is frequently used in studies of instrument acoustics (Iverson and Krumhansl, 1993; McAdams et al., 1995; Sandell, 1995; Lakatos, 2000; Schubert et al., 2004). However, this measure is not often used in the study of speaking voice acoustics, where due to the special nature of speech, resonance frequency measures obtained from LPC analysis or spectral peaks measured directly from the output spectral tend to be used. However, in female singing voices, it is difficult to obtain acoustic measures that directly correlate to actual resonance frequencies. As pitch increases, the increasingly wide spacing of harmonics makes it unlikely that these resonance peaks will be represented precisely in the acoustic output spectrum, particularly at fundamental frequency above 350 Hz (Monsen and Engebretson, 1983). For this reason, when vowel is constant, spectral centroid may provide a better measure of the center of spectral mass than those typically used for speech and is a measure that can be employed across the wide range of frequencies that span the female singing voice range. The current study employed three spectral centroid measures, spectral centroid from 0 to 8 kHz, spectral centroid from 0 to 2 kHz, and spectral centroid from 2 to 8 kHz. Spectral centroid from 0 to 8 kHz provides a measure that mathematically corresponds to the center of mass below 8000 Hz and is influenced both by the location of resonance frequencies and spectral slope. Spectral centroid from 0 to 2 kHz provides a measure of the center of mass in the range of the vowel formants. Spectral centroid from 2 to 8 kHz provides a measure of the center of mass in the upper frequencies, a range that has been shown to provide cues to classical singing voice categories when F1 and F2 frequencies are held constant (Berndtsson and Sundberg, 1995) and has been shown to correlate more strongly than other measures to voice category (Frič and Pavlechová, 2018). All spectral centroid measures were calculated after Sandell (1995) using the formula:

(3) $$\frac{\sum_{k=1}^{N}e_{k}\,f_{k}}{\sum_{k=1}^{N}e_{k}}$$

where *e* is the vector of spectral amplitude data points and *f* is the vector of spectral frequency data points.

#### Energy Ratio

The singing power ratio (SPR) has been shown to correlate with some aspects of the perception of singing vocal timbre (Omori et al., 1996; Watts et al., 2003). SPR, which is also the Hammarberg Index (Hammarberg et al., 1980) multiplied by −1, is calculated by measuring the ratio of power of the strongest harmonic in the 2–4 kHz frequency range to the power of the strongest harmonic in the 0–2 kHz frequency range and converting to decibels (dB). SPR provides a measure of the degree to which maximum power changes from one frequency range to another and, therefore, provides a measure of output spectral slope independent of the frequency location of spectral peaks. SPR is a difficult measure to employ for noise vocoded stimuli, so the current paper utilized a related output spectral slope measure that does not rely on the measurement of the amplitude of a specific harmonic, the energy ratio (ER). ER was calculated as the ratio of the total energy in the 0–2 kHz range to the total energy in the 2–8 kHz range in dB. Comparison of SPR and ER for the non-vocoded stimuli revealed high positive correlations between these two variables (*R* = 0.990–0.999, *p* < 0.001), suggesting that ER is an appropriate substitute for SPR in this study.

## Results

### Reliability Analysis

Due to the length of the current study, listeners heard each stimulus once only, so it was not possible to conduct analyses of intra-rater consistency. Inter-rater consistency was measured through computation of intraclass correlation coefficients (ICCs) for each condition using a two-way, random-effects model. Because this study used responses averaged across all listeners (see section Multidimensional Scaling Analysis), the type of ICC employed was “the mean of *k* raters.” This type of inter-rater ICC evaluated the consistency of mean responses and ranged from 0.930 to 0.958 (Table 3). High inter-rater ICCs based on the consistency of mean responses should not be misinterpreted as suggesting that each individual rater was consistent with all other raters, only that the average was consistent. Single-rater ICCs evaluate how reliable each listener is compared to the other listeners. Single-rater ICCs were poor, ranging from 0.315 to 0.437. Poor single-rater ICCs were not unexpected since each stimulus pair was played only once (see section Multidimensional Scaling Analysis).

**TABLE 3:** ICC estimates of inter-rater consistency and their 95% confidence intervals based on a mean-rating (*k* = 29) 2-way random-effects model for all six conditions.

Condition	ICC	95% Confidence Interval	*F*(14, 392)	*p*
C4 Not vocoded	0.947	0.919–0.969	18.906	<0.001
C4Vocoded	0.930	0.893–0.959	14.327	<0.001
B4Not vocoded	0.954	0.929–0.973	21.715	<0.001
B4Vocoded	0.952	0.926–0.972	20.818	<0.001
F5Not vocoded	0.958	0.935–0.975	23.550	<0.001
F5Vocoded	0.943	0.913–0.967	17.650	<0.001

### Multidimensional Scaling Analysis

**FIGURE 3:**
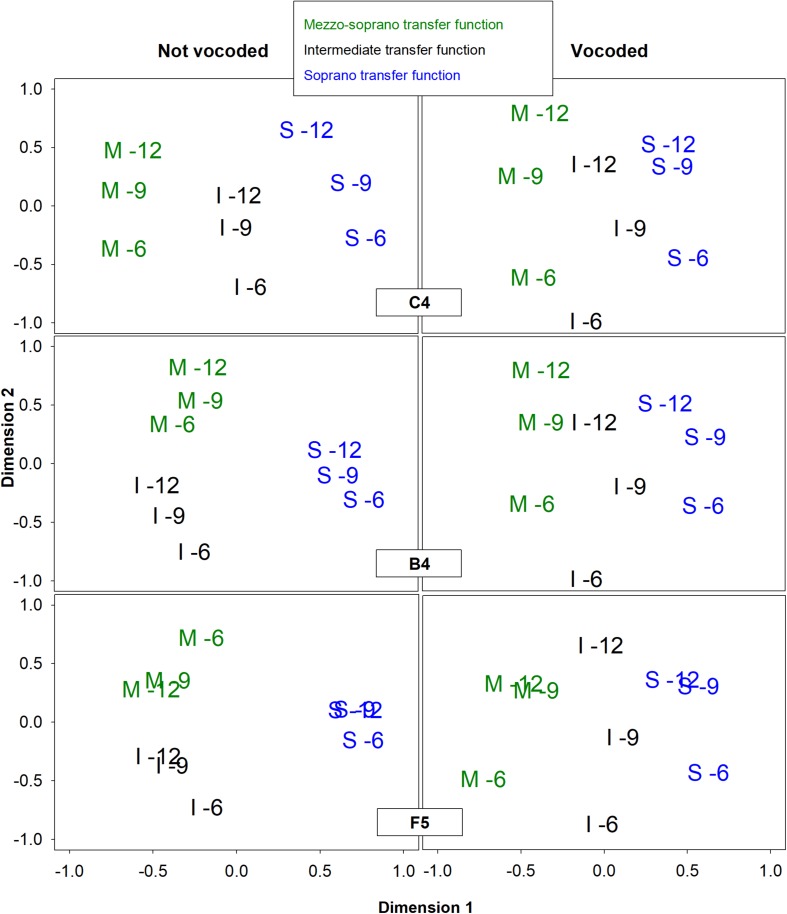
Two-dimensional representations of the MDS perceptual spaces for non-vocoded and vocoded stimuli at pitches C4, B4, and F5. Graph symbols indicate the vocal tract transfer function category of Mezzo-soprano (M), Intermediate (I), or Soprano (S) followed by the glottal excitation source slope.

Six multidimensional scaling (MDS) analyses were conducted to determine the perceptual dimensionality of the vocal stimuli based on the average responses of listeners to the experimental task. Average responses were used due to the fact that participants heard each stimulus pair once only. By using data representing how an average listener might respond, the effect of response variability including mistakes due to fatigue or lapses of attention were minimized. Separate PROXSCAL analyses were performed for each condition, not vocoded and vocoded, at each pitch, C4, B4, and F5, using IBM SPSS Statistics version 24.0 (IBM, Armonk, NY, United States). All PROXSCAL analyses used ordinal distance measurements with ties allowed and Euclidian metrics. As suggested by Borg and colleagues (Borg et al., 2013), the following model options were used: (Migirov et al., 2009) stress convergence = 0.000001, (Limb and Roy, 2014) minimum stress = 0.0001, (Jiam et al., 2017) maximum iterations = 1000, and an initial model configuration set to multiple random starts = 5000. Because there were only 9 stimuli in each condition, all MDS models were restricted to 2 dimensions. Higher dimensional models would likely have resulted in nearly perfect, but meaningless, fit. A Kruskal’s stress type 1 of 0.2 is considered to be a poor fit (Kruskal and Wish, 1978). Model fit was evaluated through analysis of Kruskal’s Stress Type 1 (Kruskal, 1964), a measure of how well the MDS solution fits the actual data, and analysis of the amount of dispersion accounted for (DAF), a measure of the variance accounted for by the MDS solution (Borg et al., 2013). Analysis of Stress Type 1 scree plots (Figure 3) revealed that for most conditions the best and most parsimonious fit was achieved with 2 dimensions. A Kruskal’s stress type 1 of 0.2 is considered to be a poor fit [27]. Analysis of DAF revealed that the 2-dimensional solutions accounted for over 98% of the variance in all conditions. Solutions for all 6 conditions are presented graphically in Figure 3. In this figure, as well as throughout the paper, the stimuli are labeled in a manner that indicates the synthesis parameters used to create the stimuli with a letter indicating the vocal tract transfer function (M = mezzo, I = intermediate, and S = light coloratura soprano) and a number indicating the excitation source spectral slope (glottal source spectral slope + lip radiation) in dB/octave (6, 9, and 12). PROXSCAL uses proximity matrices to find a default least squares solution that is arbitrary in orientation and rotationally invariant. All MDS solutions in Figure 3 are presented in their default orientation except for condition B4 not vocoded, which was rotated counterclockwise 45%, and condition F5 vocoded, which was rotated 25% clockwise. Conditions B4 not vocoded and F5 vocoded displayed dimensionality that was nearly identical to other conditions; however, the dimensional organization was off-axis. Because the dimensionality of MDS solutions is arbitrary and rotationally invariant, rotation of aggregate MDS data points is allowed and is a common practice (Peay, 1988; Giguère, 2006; Borg et al., 2013). Without such rotation, it would not have been possible to compare solutions across conditions (Peay, 1988) or to conduct statistical analyses such as correlations or regressions (Borg et al., 2013). The rotation necessary to align these two off-axis conditions was determined by the following process: (Migirov et al., 2009) matrix rotation was applied using an initial ballpark direction and degree of rotation derived from visual inspection of the MDS plots and (Limb and Roy, 2014) a “brute force” procedure was applied using increments or decrements of 5 degrees until the alignment of data points for these two MDS solutions agreed with those obtain from other conditions.

#### MDS Results

For both non-vocoded and vocoded conditions at the pitch C4, dimension 1 separated stimuli according to vocal tract transfer functions M, I, and S, while dimension 2 organized the stimuli according to excitation source spectral slope (glottal source spectral slope + lip radiation). The MDS solutions for vocoded stimuli at the higher pitches, B4 and F5 looked very similar to those obtained at the lower pitch, C4. Dimension 1 organized the stimuli according to vocal tract transfer function, M, I, and S, while dimension 2 organize the stimuli according to excitation source spectral slope. On the other hand, the MDS solutions for the non-vocoded stimuli at B4 and F5 looked quite different from those obtained at C4. Dimension 1 did not separate the 3 vocal tract transfer functions, but instead separated the soprano vocal tract transfer function from both the intermediate and mezzo-soprano transfer functions. Dimension 2 for the non-vocoded stimuli organized the stimuli according to excitation source spectral slope; however, there was a reversal in order for the mezzo-soprano stimuli at pitch F5.

### Relationship of MDS Dimensions to Acoustic Variables

Stepwise regression analyses were performed to see which acoustic measure or combination of measures best predicted each MDS dimension for all conditions. These stepwise regression analyses resulted in models with either 1 or 2 significant predictors. To test collinearity, variance inflation factors (VIFs) (Hocking and Pendelton, 1983; Craney and Surles, 2002) were computed for all 2-predictor models. All 2-predictor models generated VIFs of less than 1.5, with all but one generating VIFs of less than 1.2, indicating that regression coefficients were not likely inflated due to collinearity.

#### Acoustic Correlates of Dimensions 1 and 2 for Non-vocoded and Vocoded Stimuli at Pitch C4

Stepwise regression analyses were conducted for both the non-vocoded and vocoded stimuli at pitch C4. The results of the stepwise regression analyses are presented in Table 4.

**TABLE 4:** Prediction of MDS dimensions by acoustic variables using forward regression for non-vocoded and vocoded stimuli at pitch C4.

Non-vocoded stimuli parameter	Dimension 1 *R*^2^ = 0.944		Dimension 2 *R*^2^ = 0.976	
		
	β	*P*	β	*P*
Centroid 0–8 kHz				
Centroid 0–2 kHz	1.027	<0.001		
Centroid 2–8 kHz			–0.303	0.008
ER	–0.655	0.001	–0.784	<0.001

**Vocoded stimuli parameter**	**Dimension 1 *R*^2^ = 0.863**		**Dimension 2 *R*^2^ = 0.853**	
		
	**β**	***P***	**β**	***P***

Centroid 0–8 kHz			–0.923	<0.001
Centroid 0–2 kHz	0.874	0.001		
Centroid 2–8 kHz				
ER	–0.607	0.008		

The results of stepwise regression analyses for non-vocoded stimuli at pitch C4 suggest that the 2 significant predictors of dimension 1 were spectral centroid from 0-2 kHz and ER (*R*^2^ = 0.944), with spectral centroid from 0 to 2 kHz being the strongest predictor. The 2 significant predictors of dimension 2 were ER and spectral centroid from 2 to 8 kHz (*R*^2^ = 0.976), with ER being the strongest predictor.

The results of stepwise regression analyses for vocoded stimuli at pitch C4 suggest that, as with the non-vocoded stimuli, the 2 significant predictors of dimension 1 were spectral centroid from 0 to 2 kHz and ER (*R*^2^ = 0.863), with spectral centroid from 0 to 2 kHz being the strongest predictor. However, unlike the stepwise regression results for non-vocoded stimuli, the significant predictor of dimension 2 for vocoded stimuli at pitch C4 was spectral centroid from 0 to 8 kHz (*R*^2^ = 0.853).

#### Acoustic Correlates of Dimensions 1 and 2 for Non-vocoded and Vocoded Stimuli at Pitch B4

Stepwise regression analyses were conducted for both the non-vocoded and vocoded stimuli at pitch B4. The results of the stepwise regression analyses are presented in Table 5.

**TABLE 5:** Prediction of MDS dimensions by acoustic variables using forward regression for non-vocoded and vocoded stimuli at pitch B4.

Non-vocoded stimuli parameter	Dimension 1 *R*^2^ = 0.943		Dimension 2 *R*^2^ = 0.621	
		
	β	*P*	β	*P*
Centroid 0–8 kHz			–0.788	0.012
Centroid 0–2 kHz	0.853	<0.001		
Centroid 2–8 kHz				
ER	–0.502	0.002		

**Vocoded stimuli parameter**	**Dimension 1 *R*^2^ = 0.985**		**Dimension 2 *R*^2^ = 0.979**	
		
	**β**	***P***	**β**	***P***

Centroid 0–8 kHz				
Centroid 0–2 kHz	1.001	<0.001	–0.262	0.006
Centroid 2–8 kHz				
ER	–0.553	<0.001	–0.880	<0.001

The results of stepwise regression analyses for non-vocoded stimuli at pitch B4 suggest that, as with pitch C4, the 2 significant predictors of dimension 1 were spectral centroid from 0 to 2 kHz and ER (*R*^2^ = 0.943), with spectral centroid from 0 to 2 kHz being the strongest predictor. The significant predictor of dimension 2 was spectral centroid from 0 to 8 kHz (*R*^2^ = 0.621).

The results of stepwise regression analyses for vocoded stimuli at pitch B4 suggest that, as with the non-vocoded stimuli, the 2 significant predictors of dimension 1 were spectral centroid from 0 to 2 kHz and ER (*R*^2^ = 0.985), with spectral centroid from 0 to 2 kHz being the strongest predictor. The 2 significant predictors of dimension 2 were ER and spectral centroid from 2 to 8 kHz (*R*^2^ = 0.979), with ER being the strongest predictor.

#### Acoustic Correlates of Dimensions 1 and 2 for Non-vocoded and Vocoded Stimuli at Pitch F5

Stepwise regression analyses were conducted for both the non-vocoded and vocoded stimuli at pitch F5. The results of the stepwise regression analyses are presented in Table 6.

**TABLE 6:** Prediction of MDS dimensions by acoustic variables using forward regression for non-vocoded and vocoded stimuli at pitch F5.

Non-vocoded stimuli parameter	Dimension 1 *R*^2^ = 0.848		Dimension 2 *R*^2^ = 0.854	
		
	β	*P*	β	*P*
Centroid 0–8 kHz				
Centroid 0–2 kHz	0.921	<0.001		
Centroid 2–8 kHz			–0.986	0.001
ER			0.410	0.049

**Vocoded stimuli parameter**	**Dimension 1 *R*^2^ = 0.729**		**Dimension 2 *R*^2^ = 0.819**	
		
	**β**	***P***	**β**	***P***

Centroid 0–8 kHz				
Centroid 0–2 kHz	0.854	0.003		
Centroid 2–8 kHz				
ER			–0.905	0.001

The results of stepwise regression analyses for non-vocoded stimuli at pitch F5 suggest spectral centroid from 0 to 2 kHz was a significant predictor of dimension 1 (*R*^2^ = 0.848). The 2 significant predictors of dimension 2 were spectral centroid from 2 to 8 kHz and ER (*R*^2^ = 0.854), with spectral centroid from 2 to 8 kHz being the strongest predictor.

The results of stepwise regression analyses for vocoded stimuli at pitch F5 suggest that, as with the non-vocoded stimuli, spectral centroid from 0 to 2 kHz was a significant predictor of dimension 1 (*R*^2^ = 0.729). The significant predictor of dimension 2 was ER (*R*^2^ = 0.819).

## Discussion

### Interpreting the MDS Solutions

MDS provides a means of visualizing relationships between objects in a multi-dimensional space and can serve to test structural hypotheses concerning latent constructs that affect the perception of those objects (Borg et al., 2013). While MDS dimensions sometimes correlate with measured variables, the real interest is often in visualizing how the stimuli group in space, and in the case of the current study, how this grouping might change with vocoding. In the sections that follow, the correlations between some measured acoustic variables and MDS dimensions are discussed. These correlations should not be interpreted as establishing a causal relationship, but instead should be interpreted as measurable acoustic variables that may load on the unmeasurable construct of timbre perception.

#### Pitch C4

It was hypothesized that at lower pitches, 8-channel vocoding would result in the loss of important spectral characteristics, resulting in alterations of the multidimensional perceptual space. While the MDS solutions for the non-vocoded and vocoded stimuli at the pitch C4 looked very similar, some important differences were also observed (see Figure 3). The non-vocoded stimuli clustered well based on voice category, occupying distinct spaces along dimension 1. The vocoded stimuli also tended to organize along dimension 1 based on voice category; however, they did not cluster as cleanly, with the I-12 stimulus appearing much closer in distance to the S-12 and S-9 stimuli. All stimuli were organized according to excitation source spectral slope along dimension 2.

#### Pitches B4 and F5

It was also hypothesized that at the higher pitches, B4 and F5, the wider spacing of harmonics would cause a loss of output spectral peaks in both the non-vocoded and vocoded conditions, theoretically resulting in similar MDS representations. Instead, notable differences between the MDS representations for non-vocoded and vocoded stimuli were seen (see Figure 3).

**FIGURE 4:**
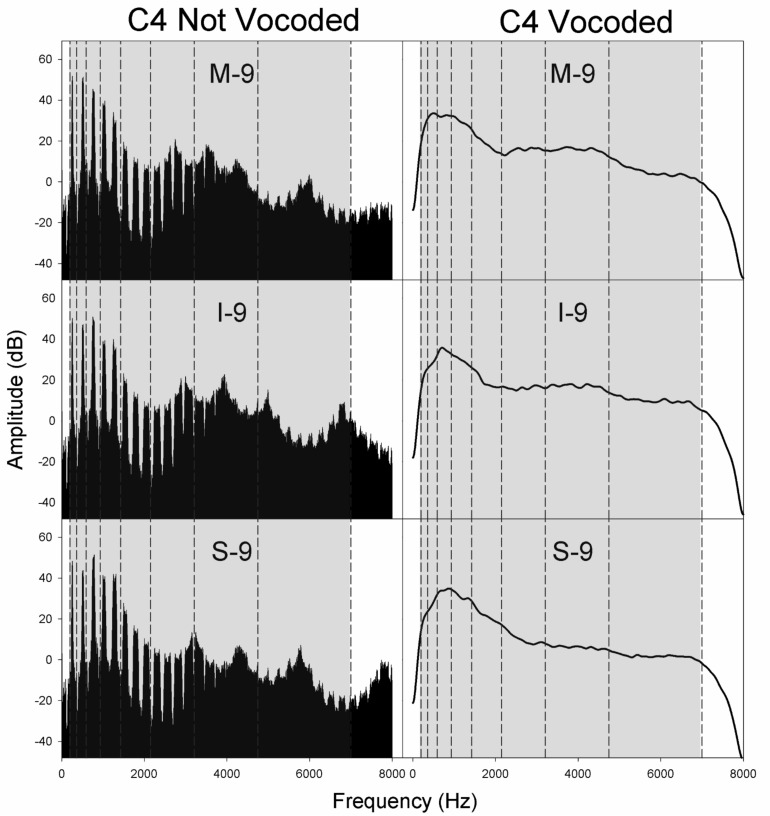
Spectra for non-vocoded and vocoded stimuli for excitation spectral slopes of −9 dB/octave at the pitch C4. The frequency range of vocoding is indicated in gray, while vocoder bands are indicated by vertical dashed black lines. Panel labels indicate the vocal tract transfer function category of Mezzo-soprano (M), Intermediate (I), or Soprano (S) followed by the glottal excitation source slope, −9 dB/octave.

**FIGURE 5:**
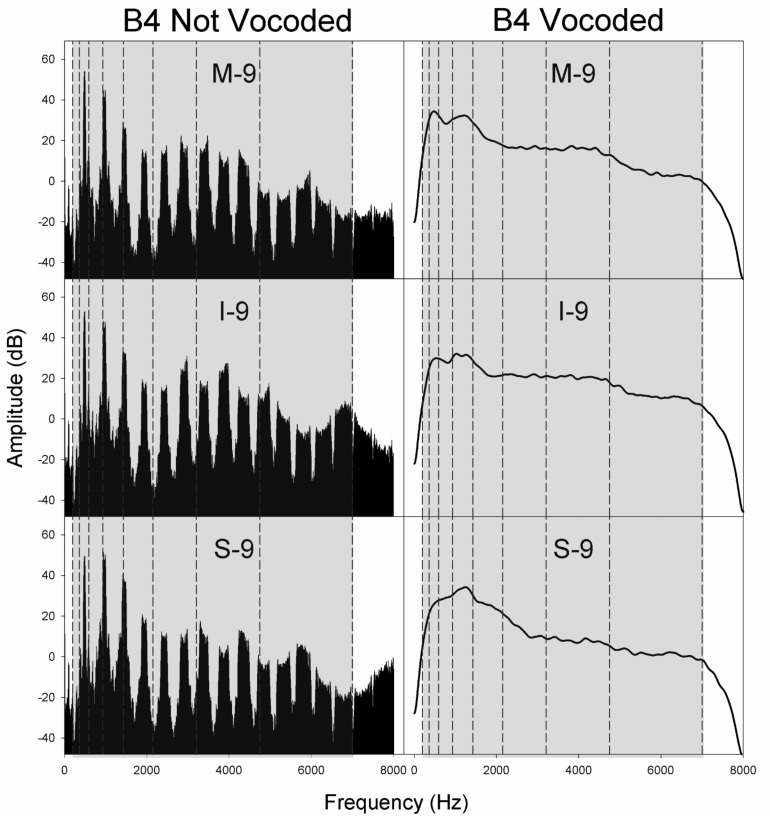
Spectra for non-vocoded and vocoded stimuli for excitation spectral slopes of −9 dB/octave at the pitch B4. The frequency range of vocoding is indicated in gray, while vocoder bands are indicated by vertical dashed black lines. Panel labels indicate the vocal tract transfer function category of Mezzo-soprano (M), Intermediate (I), or Soprano (S) followed by the glottal excitation source slope, −9 dB/octave.

**FIGURE 6:**
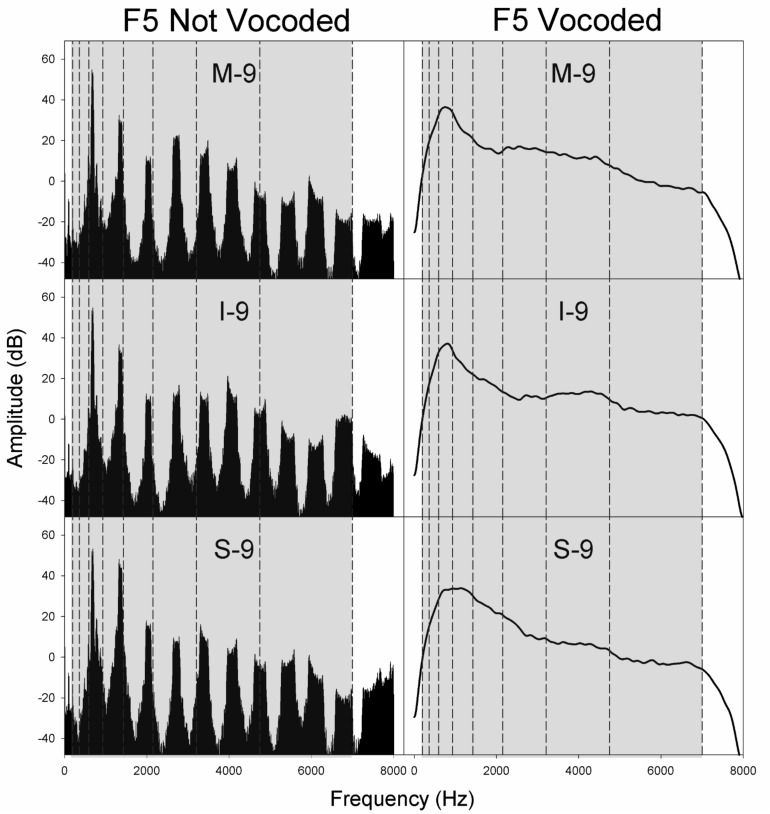
Spectra for non-vocoded and vocoded stimuli for excitation spectral slopes of −9 dB/octave at the pitch F5. The frequency range of vocoding is indicated in gray, while vocoder bands are indicated by vertical dashed black lines. Panel labels indicate the vocal tract transfer function category of Mezzo-soprano (M), Intermediate (I), or Soprano (S) followed by the glottal excitation source slope, −9 dB/octave.

As with the C4 stimuli, non-vocoded stimuli at the pitches B4 and F5 appeared distinctly clustered in the MDS space according to voice category. However, these stimuli were not distributed in the order of voice category along dimension 1. Instead, at these higher pitches (Migirov et al., 2009), the distances between mezzo-soprano and soprano stimuli were less than those observed for pitch C4 and (Limb and Roy, 2014) for some stimulus pairs, the non-vocoded mezzo-soprano and intermediate stimuli were equidistant from the non-vocoded soprano stimuli. To understand these differences, a look at the original aggregate dissimilarities may prove informative. Table 7 displays a subset of the original aggregate listener dissimilarities where stimulus pairs differed only in voice category. At the pitches B4 and F5, listeners heard mezzo-soprano and soprano stimuli as less dissimilar than they did at the pitch C4. Also, for some stimulus pairs at both B4 and F5, listeners heard mezzo-soprano and intermediate stimuli as equally dissimilar to soprano stimuli. Spectral centroid from 0 to 2 kHz was the strongest predictor of dimension 1 at pitch B4 and was the sole predictor of dimension 1 at the pitch F5. Examination of the spectral energy in the 0–2 kHz range at these higher pitches may (Migirov et al., 2009) provide clues as to why the mezzo-soprano and soprano stimuli were heard as less dissimilar and (Limb and Roy, 2014) why, in some cases, the mezzo-soprano and intermediate stimuli were heard as equally dissimilar to the soprano stimuli. Figures 4–6 display the spectra for all stimuli with a glottal excitation source of −9 dB/octave for the pitches C4, B4, and F5, respectively. These figures illustrate how under-sampling of the vocal tract transfer function due to widely spaced harmonics can lead to alterations in the perception of dissimilarity. Examination of Figures 4–6 reveals that generally, but also particularly in the area of 0–2 kHz, spectral details that were present at the pitch C4 were lost at pitches B4 and F5. This loss of spectral information could result in a smaller perceived dissimilarity between mezzo-soprano and soprano stimuli. Also, at these higher pitches, the spectra for the mezzo-soprano and intermediate stimuli from 0 to 2 kHz appear to be very similar, with the first harmonic being higher in amplitude than the second. However, for the soprano stimuli, the first harmonic is equal in amplitude to the second harmonic, possibly contributing to perception that the mezzo-soprano and intermediate stimuli were equally dissimilar to the soprano stimuli. These spectral differences may have contributed to the perceived dissimilarities presented in Table 7, which in turn generated the MDS spaces seen in Figure 3.

**TABLE 7:** Listener dissimilarity measures for glottal source excitation slopes of −6, −9, and −12 dB/octave at the pitches C4, B4, and F5.

Glottal excitation source slope (dB//Octave)	Voice category pair	Pitch		
		
		C4	B4	F5
−6	M vs. S	66.45	60.90	61.28
	I vs. S	39.00	50.62	50.21
	M vs. I	40.24	56.45	60.00
−9	M vs. S	63.90	56.21	54.72
	I vs. S	46.03	54.66	53.14
	M vs. I	52.45	53.93	39.86
−12	M vs. S	54.17	52.55	49.72
	I vs. S	41.10	59.24	49.90
	M vs. I	48.59	50.24	35.45

*M, Mezzo-soprano; I, Intermediate; S, Soprano.*

At the pitch F5, other changes in the non-vocoded MDS space begin to emerge. Stimuli having excitation source spectral slopes of −9 dB/octave and −12 dB/octave appeared closely spaced to each other at this pitch.

At the pitches B4 and F5, the MDS spaces for the vocoded and non-vocoded stimuli revealed notable differences in organization, contrary to our hypothesis. Unlike the MDS spaces for the non-vocoded stimuli at the pitches B4 and F5, the MDS spaces for the vocoded stimuli at the pitches B4 and F5 looked similar to those seen at the pitch C4. At the pitch F5, however, the mezzo-soprano and soprano stimuli with excitation source spectral slopes of −9 dB/octave and −12 dB/octave appeared closely spaced to one another, just as in the non-vocoded MDS representation. The finding that the MDS spaces for the vocoded stimuli at pitches B4 and F5 looked similar to the MDS space at pitch C4 is somewhat unexpected and is discussed further in the section that follows.

### Effects of Pitch and Location of Vocoder Bands

The effect of vocoding on normal hearing listeners’ perception of vocal dissimilarity is likely related to several interacting factors: (a) the pitch of the non-vocoded stimuli, (b) the location of vocal tract resonances, and (c) the center frequency and bandwidth of vocoder filter bands.

Figures 4–6 display the non-vocoded and vocoded spectra for mezzo-soprano, intermediate, and soprano stimuli with an excitation source spectral slope of −9 dB/octave for the pitches C4, B4, and F5, respectively. The frequency range of vocoding is indicated in gray, while vocoder bands are indicated by vertical dashed black lines. Because spectral centroid from 0 to 2 kHz and ER were the strongest predictors of dimension 1 at pitches C4 and B4, and spectral centroid from 0 to 2 kHz was the sole predictor of dimension 1 at the pitch F5, examination of the spectral energy in the 0–2 kHz region for both non-vocoded and vocoded stimuli may prove informative.

At the pitch C4, the closer spacing of harmonics allowed for better representation of resonant peaks in the non-vocoded output spectra. Clear differences in spectral peak location of the non-vocoded stimuli, S-9, I-9, and M-9, in the 0–2 kHz range can be seen. At pitch C4, the first 3 harmonics are each located in a separate vocoder band, resulting in spectral peaks in the vocoded stimuli that seem to correspond reasonably well with those seen in the non-vocoded stimuli. Above 2 kHz, spectral peaks and valleys in the non-vocoded stimuli are located such that when vocoded, these peaks and valleys average out, creating a spectrum from 2 to 7 kHz in the vocoded stimuli that is fairly flat.

At the pitch B4, for the non-vocoded stimuli, the wider spacing of harmonics resulted in a large 1^*st*^ harmonic amplitude for I-9 and M-9, while for S-9, the 1st and 2nd harmonics are of almost equal amplitude. The fundamental is located in vocoder band 2, while the 2nd harmonic oscillates about the border between bands 3 and 4 and the 3rd harmonic oscillates about the border between band 4 and 5. The result is a strong peak in the vocoded spectrum for S-9 that is slightly higher in frequency than that of the non-vocoded S-9 stimulus. The most important effect at B4, however, occurs with I-9 and M-9. The vocoded spectra for these 2 conditions exhibit a second artifactual spectral peak that is not present in the original spectra. This is likely due to the oscillating 2nd and 3rd harmonics crossing into and out of neighboring vocoder filter bands. As with the pitch C4, in the area of 2–7 kHz, the vocoded spectra are relatively flat.

At the pitch F5, for the non-vocoded stimuli, the 1st harmonic is located in vocoder band 3. The 2nd harmonic appears to be located within vocoder band 4 but is also oscillating on the boundary with vocoder band 5. After vocoding, the resulting spectra appear to correspond reasonably well with the non-vocoded spectra in the region of 0–2 kHz. The vocoded spectra above 2 kHz are relatively flat.

The introduction of artifactual spectral peaks in the vocoded condition at pitch B4 for the I-9 and M-9 stimuli may have contributed to the unexpected MDS solutions at this pitch. However, artifactual peaks were not introduced in the vocoded condition at the pitch F5, which exhibited the same phenomenon. Because MDS spaces are only gross approximations of the perceptual space and because dimension 1 was best predicted by a weighted linear combination of both spectral centroid from 0 to 2 kHz and ER at pitch B4, but only by spectral centroid from 0 to 2 kHz at pitch F5, it is difficult to say with certainty that the extra spectral peak in the vocoded condition at pitch B4 was responsible for the differences in organization along dimension 1 between non-vocoded and vocoded stimuli, however, the possible introduction of artifactual spectral peaks, in addition to the possible loss of spectral peaks, during the vocoding process must be considered.

### Implications for Cochlear Implant Users

In the current study, there were some instances where normal hearing listeners perceived timbral differences in the non-vocoded conditions that they did not in the vocoded conditions. Conversely, there were situations where introduction of artifactual peaks in the vocoded stimuli may have resulted in normal hearing listeners perceiving timbral differences in the vocoded conditions that they could not perceive in the non-vocoded conditions. Yet, in general, the MDS solutions for non-vocoded and vocoded conditions were similar, suggesting that, for the most part, normal hearing listeners were able to extract some timbral information from the degraded vocoder signal. The degree to which this might happen for CI users will likely depend on the design of the cochlear implant as well as the pitch and resonance characteristics of the singer.

Overall, CI users have poor music perception for many reasons. Device-related factors may affect music perception, including: (a) mismatched frequency-place alignment; (b) spectral smearing as a result of channel interaction and spread of neural excitation; and (c) factors related to the signal processing strategy employed by the device (Limb and Roy, 2014), such as using monopolar vs. all-polar stimulation modes (Marozeau and McKay, 2016). Further, listener factors may limit perception. These listener factors include: (a) variable patterns of nerve survival; (b) electrode array position; and (c) residual acoustic hearing (Bierer and Faulkner, 2010; Limb and Roy, 2014; Pfingst et al., 2015). At the central processing level, there may be extensive changes in the brain as a result of auditory deprivation (Stropahl et al., 2017) as well as altered general cognitive abilities (Holden et al., 2013; Kramer et al., 2018). Therefore, even though timbral cues might be preserved by the initial cochlear implant signal processing, the extent to which each CI user can make use of these cues (i.e., perceptual weighting) may be highly variable (Winn et al., 2016).

One possible device issue that may impact the perception of vocal timbre in CI users concerns spectral slope. Some singing voice styles (Sundberg et al., 1993, 1999; Thalén and Sundberg, 2001; Stone et al., 2003; Björkner, 2008; Bourne and Garnier, 2012; Bourne et al., 2016) and singing voice registers (Sundberg and Kullberg, 1999; Sundberg and Högset, 2001; Roubeau et al., 2009) are differentiated primarily or partially by changes in glottal configuration that manifest in changes in spectral slope. Because CIs typically implement various degrees of amplitude compression, it may not be possible to detect some of the distinctions between voice styles and/or voice production types.

Given the device- and patient-related factors associated with CI use, another approach to improving music perception may be through auditory training. Several studies have shown that following training, CI users have improved their ability to discriminate musical pitch, identify melodic contours, recognize and identify familiar melodies, and identify the timbre of musical instruments (Driscoll, 2012; Galvin et al., 2012; Petersen et al., 2012; Gfeller et al., 2015; Fuller et al., 2018).

### Strengths of the Study

One strength of the study is that every participant completed all 6 MDS conditions. Thus, individual differences in the use of the scroll bar or other systematic idiosyncratic behaviors would be expected to be similar across all conditions, allowing each participant to serve as their own control. This allows for the visual comparison of MDS results across conditions.

A second strength of the study is that, while the number of stimuli were necessarily small in each condition, excitation source spectral slopes and vocal tract transfer functions spanned the range typically seen in female singers.

### Limitations of the Study

Generally, the current study suffers from the same limitations that befall all studies employing a non-experimental modeling procedure such as MDS. While MDS studies can provide useful insight into how listeners’ perceptions are organized, correlating any acoustic parameter to a dimension can be problematic. Thus, while this study found that a linear combination of acoustic variables could predict the MDS dimensions in all conditions very well, this prediction cannot be directly related to human perception, which is a complex phenomenon that likely cannot be reduced to a set of numbers derived from acoustic measurements.

Because each participant completed all conditions, time constraints resulted in several limitations. Each participant by necessity heard all stimulus pairs once only. This required the use of aggregate MDS so that the effect of any errant responses could be minimized. Thus, INDSCAL analyses could not be employed. Additionally, the number of stimuli in each condition had to be restricted to no more than 9, which limits the number of dimensions that can safely be employed in the MDS to two. Finally, additional conditions using a variety of vocoder configurations could not be employed.

The current study utilized 8-channel vocoded stimuli to assess the perceptual dimensionality of singing voice timbre. While vocoders provide a clue as to how the degraded cochlear implant signal might affect the perception of timbral dissimilarity, it cannot be assumed that these results will directly translate to the perception of timbre in cochlear implant populations for reasons highlighted in the previous section.

### Future Research

#### The Role of Context in CI Listener Timbre Perception

While the current study manipulated glottal excitation source slope and vocal tract transfer function, the purpose of the study was to test overall vocal timbre perception. In such studies of vocal timbre, variations in vowel must be kept to a minimum. Even when perceptual studies have been specifically designed to experimentally test a parameter such as voice category perception, researchers have (a) limited the stimuli to just one vowel (Cleveland, 1977; Berndtsson and Sundberg, 1995; Erickson, 2004) or (b) performed a long-time-average spectra (LTAS) over a part or the entirety of a song (Johnson and Kempster, 2011). Future research should examine vocal timbre perception and voice category perception using a variety of vowels and in a variety of contexts.

#### The Role of Vibrato in the Timbre Perception of CI Listeners

In addition to perceptual information provided by glottal excitation spectral slope and vocal tract transfer function, vibrato may play a role in the ability to hear timbral differences between voices. Vibrato emerges in a Western classical singing voice first as a coherent frequency modulated (FM) voice source which when filtered by the vocal tract produces spectral fluctuations and a secondary amplitude modulation (AM) as harmonics move into and out of vocal tract resonances. Thus, classical vibrato singing is characterized by both FM (also termed frequency vibrato) and AM (also termed amplitude vibrato).

The possible role of vibrato as a timbre cue available to CI listeners has not been well researched. While CI listeners may not be able to hear the fine structure needed to perceive frequency vibrato, the spectral fluctuations associated with vibrato across frequency bands may provide a better representation of timbre than may be available from a non-modulated vocal stimulus (McAdams and Rodet, 1988). These spectral fluctuations may also give rise to the perception of vibrato rate and/or extent, an element of timbre that may assist in the discrimination of voices. Additionally, both the frequency and extent of secondary amplitude modulations could provide salient timbral cues. Future research should examine the role that vibrato may play in timbre discrimination in both NH listeners presented with vocoded stimuli and CI listeners by Migirov et al. (2009) utilizing synthetic stimuli that vary in vibrato rate or (Limb and Roy, 2014) utilizing real singing voices. Given that training has been shown to improve music perception in CI users, the knowledge gained from such studies could be used to develop and test training strategies.

## Summary and Conclusion

Vocal tract resonance frequencies have been shown to be a cue to the perception of voice categories such as baritone, tenor, mezzo-soprano, and soprano, while changes in glottal source spectral slope are believed to be related to perception of vocal quality dimensions such as *fluty* vs. *brassy* and are associated with the production of various singing styles and singing registers. For the simulated mezzo-soprano, intermediate, and coloratura soprano voices used in this study, MDS solutions grouped stimuli according to voice category and excitation source spectral slope in all conditions. However, while stimuli tended to be grouped by voice category, such grouping did not always correlate with an MDS dimension. Excitation source spectral slope was generally represented as increasing along dimension 2; however, at the pitch F5 where widely spaced harmonics would not likely line up with vocal tract resonances well, thus obscuring some elements of excitation source spectral slope, this organization did not always hold. While it is unclear how well these timbre percepts would emerge as MDS dimensions for CI listeners, in general, these results suggest that perhaps some aspects of vocal timbre may remain and combined with other information such as vibrato rate, may provide some cues to singer identity.

## Data Availability Statement

The datasets generated for this study are available on request of the corresponding author.

## Ethics Statement

This study was carried out in accordance with the recommendations of the Institutional Review Board of the University of Tennessee, Knoxville, with written informed consent from all subjects. All subjects gave written informed consent in accordance with the Declaration of Helsinki. The protocol was approved by the Institutional Review Board of the University of Tennessee, Knoxville.

## Author Contributions

ME, PJ, MH, and KF contributed to the conception and design of the study. ME performed the MDS analyses and wrote the first draft of the manuscript. TS contributed to data analysis and interpretation. PJ, MH, and KF provided expertise concerning cochlear implants and contributed ideas for the introduction and discussion sections. KF wrote a section in the discussion section. All authors contributed to manuscript revision, read and approved the submitted version.

## Conflict of Interest

KF is now an employee of Oticon Medical, however, her contributions to this study were made while she was working at the University of Tennessee Health Sciences Center. The remaining authors declare that the research was conducted in the absence of any commercial or financial relationships that could be construed as a potential conflict of interest.
